# *Phoma* Infections: Classification, Potential Food Sources, and Their Clinical Impact

**DOI:** 10.3390/microorganisms6030058

**Published:** 2018-06-23

**Authors:** Ashely Bennett, Michelle M. Ponder, Julia Garcia-Diaz

**Affiliations:** 1 Department of Infectious Diseases, Ochsner Medical Center, New Orleans, LA 70121, USA; ashely.bennett@ochsner.org (A.B.); michelle.ponder@ochsner.org (M.M.P.); 2 Department of Internal Medicine, Ochsner Clinical School, University of Queensland, Brisbane, QLD 4072, Australia

**Keywords:** *Phoma* spp., subcutaneous mycosis, phaeohyphomycosis, food

## Abstract

*Phoma* species are phytopathogens that are widely distributed in the environment, most commonly found in aquatic systems and soil. *Phoma* spp. have the potential to be pathogenic in plants, animals and humans; the latter is a rare occurrence. However, as our immunocompromised population increases, so do the reports of these infections. Medical advances have allowed for the increase in solid organ transplantation; chemotherapies to treat malignancies; and the use of other immunosuppressive agents, which have resulted in a greater population at risk when exposed to diverse fungi including *Phoma* spp. These fungi have been isolated from water sources, food, and crops; thus acting as opportunistic pathogens when the right host is exposed. *Phoma* spp. contaminates common food sources such as potatoes and maize, a common species isolated being *Phoma sorghina*. Though there is potential for causing infection via consumption of contaminated foods, there is insufficient data detailing what levels of organism can lead to an infection, and a regulated process for detecting the organism. The spectrum of disease is wide, depending on the host, ranging from cutaneous infections to invasive diseases. Mortality, however, remains low.

## 1. Introduction

**Figure 1:**
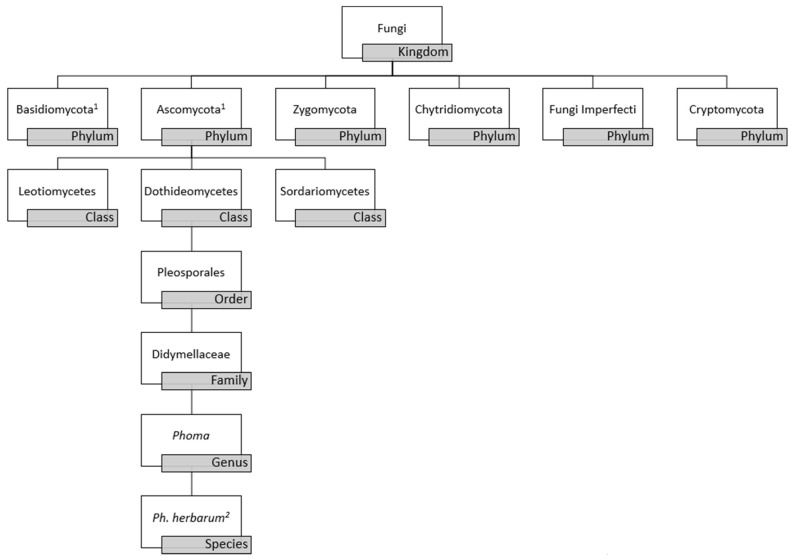
*Phoma* species schema based on current classification data utilizing morphologic and molecular characterization data. (1) Basidiomycota and Ascomycota are more closely related to one another than to other phyla. (2) Use of molecular based phylogenetic analyses has restricted the *Phoma* genus to *Phoma herbarum* sp. within family Didymellaceae.

*Phoma* is a polyphyletic genus of fungal organisms belonging to the phylum Ascomycota, class Dothideomycetes, order Pleosporales, and family Didymellaceae, as depicted in Figure 1 [1,2,3]. *Phoma* spp. was conceptualized in the 19th century by Italian mycologist Pier Andrea Saccardo (1880), and almost 100 years later updates were made to the definition and classification of the genus by Gerhard Boerema and Gerrit Bollen (1975) [4,5]. Greater than 220 species were formally recognized in the handbook, “Phoma Identification Manual” by Boerema et al. with identification determined by morphological characteristics, such as the formation of conidia (asexual spores), pycnidia (asexual fruiting bodies), and chlamydospores (enlarged, thick-walled vegetative cells within hyphae or at hyphal tips) [6,7]. *Phoma* spp. classically has been grouped in the class Coelomycetes due to such morphological features. However, this classification has been determined to be obsolete as a result of the increased use of phylogenetic analyses used to classify *Phoma* spp. Though obsolete, the term Coelomycetes is still used in the clinical setting [2]. Overall, classification of fungi as a whole is under dynamic revision due to availability of modern molecular techniques for analysis of fungi at the genomic, transcriptomic, and proteomic level. Current data suggests that all fungi may be encompassed within three phyla, thus future studies are needed to truly classify *Phoma* spp. within the kingdom fungi [8].

*Phoma* spp. constitutes a diverse group of organisms that are ubiquitous; generally found in soil, organic matter, plants, and water sources. Fungal organisms belonging to the genus *Phoma* are known to be phytopathogens, characterized by parasitic relationships with plants. *Phoma* spp. can change from opportunistic to pathogenic organisms once in contact with the appropriate host [9]. The species have been reported to be an opportunistic invasive pathogen in animals and humans. The first documented case caused by *Phoma* spp. proven mycologically and histologically in a human was a subcutaneous lesion in a post-renal transplant patient in 1973 [10]. The infections resulting from *Phoma* spp. are increasing with the advancement of medicine, primarily due to the increase in patients who are at risk due to immunosuppression.

Given the consistent rise in opportunistic fungal infections correlating to an increase in individuals who are immunosuppressed [11], food sources typically contaminated by *Phoma* spp. can pose a greater threat to humans than just causing rot in crops. Consumption of foods that *Phoma* spp. commonly contaminate can serve as fomites for invasive fungal infections. *Phoma* spp. have been known to contaminate seeds, nuts, soybeans, potatoes, bananas, sorghum, maize, kiwi berries, lemons, tomatoes, eggplants, and pomegranates [12,13,14,15,16,17,18,19,20,21,22,23,24,25,26]. *Phoma* spp. produce metabolites, which can be cytotoxic; including cytochalasin A and B, deoxaphomin, proxiphomin and tenuazonic acid [27,28,29,30]. *Phoma sorghina* produces tenuazonic acid, a mycotoxin which has been reported to cause acute toxic effects in animals, such as precancerous changes in esophageal mucosa in mice who were fed tenuazonic acid over the course of 10 months [31]. Oliveira et al. reports that production of tenuazonic acid secondary to ingestion of *Phoma sorghina* infected grains may correlate with a hemorrhagic disorder in humans known as onyalai in Brazil [19]. Given that *Phoma* spp. is a contaminant in a variety of foods and has the potential for pathogenicity, it seems that additional standardized food safety practices are warranted for individuals who are immunocompromised.

## 2. Morphology and Molecular-Based Taxonomy

Distinguishing features of the *Phoma* spp. include the ability to form asexual fruiting bodies lined with conidiophores, which are spore-producing hyphae. The colonies tend to be powdery or velvety in texture and spread, while the pigmentation can vary from greenish gray to brown [32]. *Phoma* spp. in vitro exhibit morphological features such as chlamydospores, conidia, and pycnidial conidiomata (fruiting structures that act as a means of dispersing conidia), which are unique to the genus, helping distinguish them from other dematiaceous fungi [6,33]. The isolation and growth of *Phoma* spp. best occurs at a pH close to 5.5. The growth media type aids in producing the best growth characteristics in *Phoma* spp. identification; oatmeal agar supports abundant pycnidia production and malt extract agar stimulates pigment production and crystal formation [5].

The high variability in microscopic morphology results in ambiguity in the classification of the genus, thus phenotypic characters are not always distinctive between the *Phoma* spp. The *Phoma* genus originally was considered to be within the Coelomycetes class due to the character of the conidiomata and the development of conidia by the fungi in the *Phoma* spp. Modern technologies have demonstrated that the Coelomycetes refers to an artificial class of fungi, distributing the genera and species which it represented into the three classes of the phylum Ascomycota [34]. Molecular datasets have gained popularity in re-classifying the taxonomy of the species. Molecular-based analyses that have been utilized to help delineate the *Phoma* genus include examination of nuclear rDNA sequences (ITS: internal transcribed spacer regions), fragments of the 28SnrRNA gene (LSU), the RNA polymerase II gene (rpb2), and the beta-tubulin (tub2) gene [33,35]. The extensive use of molecular-based phylogenetic analyses has restricted the *Phoma* genus to *Phoma herbarum* within the family Didymellaceae with Figure 1 demonstrating the current taxonomy. Even with molecular and morphological data, the *Phoma* genus is still taxonomically controversial.

## 3. Ecological Distribution

Due to the ubiquitous nature of fungi, *Phoma* spp. has been reported in multiple natural habitats including aquatic environments, water distribution systems, soil and air [36,37,38,39,40,41,42]. While the existence of *Phoma* spp. contamination in water systems has been well documented, recent data demonstrates they are also a contaminant of multiple food sources [13].

In a recent report, Paterson et al. discussed the role food contaminated with fungi plays in the development of opportunistic infections [18]. Their report details a database of potentially pathogenic filamentous fungi that have been isolated from food/crops in which *Aspergillus* spp., *Fusarium* spp., and *Mucor* spp. were isolated from a variety of foods such as gingerbread, soy products, pasteurized beverages, tea, wheat, butter, cinnamon, cashew nuts, cauliflowers, maple syrup and sugarcane [18]. *Aspergillus* spp. were reported to be present in the majority of food samples reviewed, including cereals, dairy products, nuts, vegetables, and fruit. Foods which are grown in close proximity to soil appear to be more contaminated, given that soil is a known source of pathogenic fungi. *Phoma sorghina* has been noted as a pathogenic organism involved with food contamination in bananas and sorghum [18]. *Phoma* spp. are fungal pathogens of potatoes, typically causing rot or gangrene. The specific species isolated from potatoes include *Phoma foveata*, *Phoma exigua* var. *exigua* and *Phoma eupyrena* [43].

The exposure to *Phoma* spp. in food sources varies globally. Adekoya et al. investigated the occurrence of fungi and mycotoxins in maize-based beer known as *umqombothi* in South Africa [21]. The beer samples analyzed via PCR in combination with 16S gene sequencing method revealed the presence of *Aspergillus*, *Penicillum*, *Saccharomyces* and *Phoma* genera. The total mean fungal load was 3.66 × 10^5^ CFU/mL, which exceeded the permissible limit, 1 × 10^4^ CFU/g, of fungi in ready-to-eat foods determined by the US Food and Drug Administration [44]. *Phoma sorghina* was isolated in 62% of the beer samples, with an incidence of 23% and a mean fungal load of 2.40 × 10^6^ CFU/mL [21]. The presence of *Phoma sorghina* may correlate with the use of raw materials, such as sorghum malt, used in the production of *umqombothi* [21]. The occurrence of mycotoxins was also investigated in the study, with the most prominent being deoxynivalenol, typically associated with *Fusarium verticilliodes* [21].

*Phoma sorghina* has been reported as a common fungal contaminant in sorghum grain production [45,46,47]. Sorghum is a cereal grain, which is ranked as one of the top five most important and consumed cereal crops in the world [19,48]. Oliveira et al. surveyed 100 samples of sorghum grains in Brazil to assess the production of tenuazonic acid (a mycotoxin) of *Phoma sorghina* strains during sorghum development [19]. The maturity of sorghum consists of four stages, and *Phoma* spp. was the most prevalent genus isolated. There were 104 *Phoma* spp. isolates discovered during the analysis, and all were identified as *Phoma sorghina* [19]. A positive correlation, though not statistically significant, was found between the frequency of *Phoma sorghina* and occurrence of tenuazonic acid; the highest average level of tenuazonic acid, 440.5 µg/kg, was observed during the fourth stage of maturity when *Phoma sorghina* reached its greatest frequency of 87.4% [19]. Though traditionally considered nuisance organisms in water sources and food/crops, fungi have the potential to be opportunistic pathogens in certain populations, especially the immunocompromised who utilize and consume the contaminated water and crops.

The natural habitats of opportunistic fungal pathogens are outside the host; therefore, we need to understand their ecology and routes of transmission. As exemplified in the few reports listed, these pathogens are quite ubiquitous in all environments.

## 4. Clinical Significance

In many documented clinical case reports, patients report trauma or immunosuppressive drug use. The first reported human case of an infection caused by a *Phoma* spp. dates back to 1956; although due to taxonomic changes, this fungal pathogen may not currently belong to the *Phoma* genus [49]. The next case reported is that of a young Canadian farmer with skin lesions on her lower extremity, which resolved with treatment [50]. The first immunocompromised host was reported by Young et al. in a patient who had undergone kidney transplantation; her infection was fully resolved [10]. A comprehensive review of the literature revealed 32 cases, as depicted in Table 1, with some possibly needing reassessment given the newest taxonomy. Of these cases, the age range is from one month old to 77 years old with a total of three pediatric cases (9.4%) [51,52,53]. Most of the cases were skin injuries ranging from superficial to deep trauma and comprising 22/32 subjects (69%) [10,50,51,53,54,55,56,57,58,59,60,61,62,63,64,65,66,67]. Five infections (16%) were eye related, due to either trauma or contact lens wear [68,69,70,71,72]. Three cases (9.4%) involved the lung [49,72,73]; one was an onychomycosis [74]; and one was an invasive rhinosinusitis [52].

*Phoma* spp. identification remains controversial and difficult at times and, as noted in most of the cases reported, the organism is labeled as *Phoma* spp. only and no speciation is noted; 17/32 (53%). The other species reported include *Phoma hibernica, Phoma cava* (2), *Phoma oculo hominis, Phoma eupyrena, Phoma minutispora, Phoma minutella, Phoma sorghina* (2), *Phoma exigua, Phoma glomerata, Phoma herbarum, and Phoma insulana*. Three cases included polyfungal infections with other somewhat rare fungal organisms present; the most devastating case being one with *Phoma* spp. and *Acremonium* in an infant with invasive rhinosinusitis. However, overall mortality was low at 2/32 (6.3%) deaths when compared to mortality from other common fungal infections. The two patients in the reported cases were highly immunosuppressed due to chemotherapy—one with acute myeloid leukemia and the other with acute lymphoblastic leukemia [52,73]. Immunosuppression was mostly due to oral steroids, chemotherapy, diabetes, and other immunosuppressives in the setting of transplantation. Infections in the immunocompetent host were usually due to trauma or another type of inoculation as seen in contact lens wearers. Although pathogenic fungi has been isolated from multiple food/crop sources, there has not been a direct correlation in the reported cases; however, this correlation may be difficult to establish. We need to remain vigilant of the ubiquitous presence of fungi and the exposure to our patients. 

## 5. Management

The mainstay of management is surgical resection of infected tissues whenever possible. Of the 32 infections reported in the literature (Table 1), topical and oral/intravenous antifungal agents were used. Topical agents such as clotrimazole and miconazole were used in most of the cutaneous infections; intravitreal amphotericin B or voriconazole in the ocular infections. Systemic therapy was used in patients with infections other than cutaneous disease and if their immune status was compromised. Antifungal agents used included amphotericin B, ketoconazole, itraconazole, voriconazole and posaconazole; oral griseofulvin was used prior to the approval of azoles. Excision of cysts, nodules and other skin lesions without antifungal treatment usually is curative in the immunocompetent host; the immunocompromised usually requires concurrent systemic antifungal treatment [76].

In vitro antifungal susceptibility of pathogenic fungi is important information for the clinician when selecting the appropriate antifungal drug and deciding on the route of administration. For the filamentous fungi, species-specific breakpoints have only been proposed for a limited number of fungal species and clinical breakpoints are lacking for most emerging mold pathogens. Valenzuela et al. reported one of the most comprehensive studies aiming at determining the distribution of the Coelomycetes in clinical samples by phenotypic and molecular characterization and in vitro antifungal susceptibility pattern of nine antifungal agents (Table 2) [34,77]. Antifungal testing showed that terbinafine, echinocandins (caspofungin, micafungin and anidulafungin) and amphotericin B were the most active against *Phoma* spp. (7) and *Phoma herbarum* (10) with a minimum inhibitory concentration (MIC) range of <0.03. Azole susceptibility varied and flucytosine showed poor antifungal activity (MIC range 0.5–16; MIC_90_ 4–16). Another report by Sutton et al. had similar results with amphotericin B and itraconazole being the most active and fluconazole exhibited an MIC 32 [52]. Everett et al. reported fluconazole and flucytosine resistance in an infection in a renal transplant patient [63]. Treatments are not well established for these fungi due to the lack of clinical breakpoints and the difficulty of performing antifungal susceptibility testing.

## 6. Discussion

*Phoma* spp. remains a taxonomically controversial genus; morphological and molecular data has been generated, but many questions remain. Molecular tools will aid in the identification and classification or re-classification of the *Phoma* complexities. Infections due to *Phoma* spp. remain low when compared to other fungi. However, the majority of these cases (13/32; 41%) were reported in the literature over the last two decades. *Phoma* spp. are found in water distribution systems, public bathrooms and other public areas such as swimming pools, bodies of water, plants, soil, air and food. This wide distribution allows for high exposure in patients who are both immunocompetent and immunocompromised. Inoculation through trauma is common in the immunocompetent but exposure to the immunocompromised is harder to elucidate and subsequently prevent. Perhaps, new guidelines need to be instituted for filamentous and yeast monitoring of water distribution systems, given our growing immunocompromised population.

In addition to surveillance of water distribution systems, healthcare providers and individuals who are immunosuppressed should also be aware of, and possibly monitor, specific foods for fungal contaminants. Analogous to water sources, foods and crops similarly pose a risk of developing opportunistic fungal infections in immunocompromised patients as fungal contaminants in food have the potential to be pathogenic. In a 10-year surveillance of fungal contamination of food within a hematological unit, a protocol of food management was analyzed, revealing that filamentous fungi were isolated from 37/456 (8.1%) types of food [78]. Processed cheeses, fruit and honey were the food types with the highest numbers of samples that tested positive for filamentous fungi. Of the 42 genera isolated, *Penicillum* spp. (22/42), *Aspergillus* spp. and *Cladosporium* spp. were the most frequent genera isolated [78]. *Phoma sorghina* has similarly been isolated from similar types of food such as sorghum and bananas [18]. The potential for fungal infection certainly exists, given fungal pathogens have been isolated from an assortment of ingested products. In order to better assess which foods pose a risk, molecular detection of fungal organisms, which has become more prevalent in identifying fungal species, could be used to detect fungal pathogens in food sources consumed by high-risk individuals in a timely manner. This type of monitoring would be more relevant for foods that have not been exposed to high temperature, long before digestion. This form of target monitoring will only be valuable if viable fungal specimens can ultimately be isolated.

As technology advances, a clearer delineation of *Phoma* spp. versus non-*Phoma* spp. fungal organisms will lead to timelier identification and possibly improved treatment. Education about the hazards of exposure to *Phoma* spp. for susceptible populations as well as for medical facilities that treat such patient populations will hopefully lead to decreasing the incidence of filamentous fungal infections in the at-risk populations.

## Figures and Tables

**Table 1:** Infections Caused by *Phoma* spp. in Humans.

Isolated Fungus	Gender/Age	Source/History	Immunosuppression	Treatment/Outcome	Reference
*Phoma* spp.	N/A	Pulmonary	N/A	N/A	Janke, D. et al. 1956 [49]
*Phoma hibernica*	F/22	Skin (deep leg)	Topical steroids	Oral griseofulvin/clinical improvement	Bakerspigel, A. 1970 [50]
*Phoma* spp.	F/42	Skin (deep heel)	Azathioprine; prednisone; s/p renal transplant	Debridement/resolved	Young, N.A. et al. 1973 [10]
*Phoma cava*	M/4	Skin (superficial ear)	Otherwise healthy	Oral griseofulvin; corticosteroid/resolved	Gordon, M.A. et al. 1975 [53]
*Phoma oculo hominis*	N/A	Eye (Corneal ulcer)	Otherwise healthy	N/A	Punithalingam, E. 1976 [75]
*Phoma cruris- hominis*	F/?	Subcutaneous	N/A	N/A	Punithalingam, E. 1979 [54]
*Phoma eupyrena*	M/18 mos.	Skin (perioral lesions)	Otherwise healthy	Clotrimazole; 15% zinc oxide paste; Dimethicone/resolved	Bakerspigel, A. et al. 1981 [51]
*Phoma minutispora* *Phoma minutispora*	M/18 M/20	Skin (face) Skin (neck)	Typhoid fever Oral steroids	Topical clotrimazole/resolved Topical clotrimazole/resolved	Shukla, N.P. et al. 1984 [55]
*Phoma minutella*	M/75	Skin (deep foot) Farmer from Dominican Republic	Steroid therapy Diabetes mellitus	Debridement; amputation for secondary gangrene/resolved	Baker, J.G. et al. 1987 [56]
*Phoma sorghina* *Phoma sorghina*	M/24 M/19	Skin (face, neck, hands) Skin (face)	Otherwise healthy Otherwise healthy	Topical miconazole/resolved Topical miconazole/resolved	Rai, M.K. 1989 [57]
*Phoma* spp.	F/24	Pulmonary (lung mass)	Acute Lymphocytic Leukemia; chemotherapy	Left lower lobectomy Amphotericin B/resolved	Morris, J.T. et al. 1995 [72]
*Phoma* spp.	M/45	Skin (deep/hands)	Otherwise healthy	Itraconazole; ketoconazole/clinical improvement	Hirsh, A.H. et al. 1996 [58]
*Phoma* spp.	F/24	Skin (deep face)	Topical steroids	Ketoconazole/resolved	Rosen, T. et al. 1996 [59]
*Phoma cava*	M/63	Skin (deep hand)	Pulmonary sarcoidosis; oral steroids	Amphotericin B; itraconazole/resolved	Zaitz, C. et al. 1997 [60]
*Phoma* spp. *Phoma* spp.	M/49 M/53	Skin (plantar; foot) Skin (plantar; foot) [Both *Phoma* and *Scopulariopsis brevicaulis* grew from the strateum corneum]	Atopic dermatitis Atopic dermatitis	Topical bifonazole and ketoconazole/No improvement; lost to follow up Topical bifonazole and ketoconazole/no improvement; lost to follow up	Arrese, J.E. et al. 1997 [61]
*Phoma* spp.	M/77	Skin (deep)	Otherwise healthy	Itraconazole/resolved	Oh, C.K. et al. 1999 [62]
*Phoma* spp.	M/72	Eye (keratitis) Globe trauma	Otherwise healthy	Debridement; keratectomy	Rishi, K. et al. 2003 [68]
*Phoma* spp.	F/50	Skin (deep hand)	s/p renal transplant	Surgical debridement; amphotericin B/resolved	Everett, J.E. et al. 2003 [63]
*Phoma* spp.	M/19	Skin (deep face)	N/A	Amphotericin B	Suh, M.K. 2005 [64]
*Phoma exigua*	M/68	Pulmonary	Acute myeloid leukemia; Diabetes mellitus	Amphotericin B; left pneumonectomy/death	Balis, E. et al. 2006 [73]
*Phoma glomerata*	M/32	Eye (endophthalmitis) Retinal detachment surgery after penetrating globe injury	None noted	Amphotericin (intravitreal); voriconazole (intravitreal)/resolved	Errera, M.H. et al. 2008 [69]
*Phoma herbarum*	F/36	Nail, toe [*Phoma herbarum, Chaetomium globosum*, and *Microascus cinereus* were isolated]	Otherwise healthy	Allylamine; sertaconazole/resolved	Tullio, V. et al. 2010 [74]
*Phoma* spp.	M/69	Skin (ganglion cysts on wrist, forearm)	Diabetes mellitus	Oral itraconazole; surgical excision/resolved	Vasoo, S. et al. 2011 [65]
*Phoma* spp.	F/1 mo.	Sinus (invasive rhinosinusitis) [*Phoma* and *Acremonium* spp. were isolated]	Acute lymphoblastic leukemia; s/p chemotherapy	Amphotericin B; posaconazole; voriconazole; debridement/death with progressive rhinocerebral extension	Roehm, C.E. et al. 2012 [52]
*Phoma* spp. *Phoma* spp.	M/45 M/48	Skin (deep knee) Skin(deep knee)	Diabetes mellitus; s/p liver transplant s/p renal transplant; s/p pancreas transplant	Oral ketoconazole; surgical excision Oral itraconazole; surgical excision	Schieffelin, J.S. et al. 2014 [66]
*Phoma* spp.	F/79	Eye (keratitis) (Risk factor: used contact lenses)	Otherwise healthy	Oral itraconazole; amphotericin eye (intravitreal); keratoplasty/resolved	Kumar, P. et al. 2015 [70]
*Phoma* spp.	F/59	Eye (corneal ulcer and abscess) (Risk factor: used contact lenses)	Otherwise healthy	Amphotericin B; (intravitreal); keratoplasty	McElnea, E. et al. 2015 [71]
*Phoma insulana*	M/79	Skin (deep foot) Foot laceration which evolved over 27 years compatible with chromo- blastomycosis	Chronic alcoholism, smoker	None/lost to follow up	Hernández-Hernández, F. et al. 2018 [67]

**Table 2:** Results of in vitro Susceptibilities of Systemic Antifungals to *Phoma* spp.

Organism	Value for the Drug (Microgram/mL)												
	AMB ^1^	5-FC	ITC	VRC	FLC	PSC	KTC	TRB	MFG	CFG	AFG	MON	NAT
Valenzuela-Lopez, N. et al. 2017 [11]													
*Phoma* spp. Range ^2^ MIC_90_	0.03–4 0.25	0.5–16 4	0.03–2 0.5	0.03–2 1	-- --	0.03–1 0.5	-- --	≤0.03 0.03	≤0.03 0.03	≤0.03 0.03	≤0.03 0.03	-- --	-- --
*Ph. herbarum* Range MIC_90_	0.12–2 1	0.5–16 16	0.25–4 1	0.06–4 1	-- --	0.12–1 1	-- --	≤0.03 0.03	0.03–0.06 0.06	0.03–0.12 0.12	0.03–0.12 0.06	-- --	-- --
Sutton, D.A. 1999 [48]													
*Phoma* spp. MIC HIBreakpoints ^3^	1 <1 = S, >2 = R	16 <16 = S, >32 = R	16 <0.5 = S, >1 = R	--	32 <32 = S, >64 = R	--	8 <8 = S, >16 = R	--	--	--	--	8 <8 = S, >16 = R	32 <32 ^4^

^1^ Abbreviations are as follows: AMB: amphotericin B, ITC: itraconazole, 5-FC: 5-fluorocytosine, VRC: voriconazole, FLC: fluconazole, POSA: posaconazole, KTC: ketoconazole, TRB: terbinafine, MFG: micafungin, CFG: caspofungin, AFG: anidulafungin, MON: miconazole, NAT: natamycin/pimaracin. ^2^ Range: MIC_90_: minimum inhibitory concentration of drug that inhibited 90% of isolates, MIC: minimum inhibitory concentration, --: Not performed. ^3^ Breakpoint: chosen concentration of an antibiotic which defines whether a species of bacteria is susceptible or resistant to the antibiotic, S = susceptible, R = susceptible. ^4^ Values < 32 presumed susceptible.

## References

[1] Parr C.S., Wilson N., Leary P., Schulz K.S., Lans K., Walley L., Hammock J.A., Goddard A., Rice J., Studer M. (2014). The Encyclopedia of Life Vol.2: Providing Global Access to Knowledge about Life on Earth. Biodivers. Data J..

[2] Valenzuela-Lopez N., Cano-Lira J.F., Guarro J., Sutton D.A., Wiederhold N., Crous P.W., Stchigel A.M. (2018). Coelomycetous Dothideomycetes with emphasis on the families Cucurbitariaceae and Didymellaceae. Stud. Mycol..

[3] Jones M.D.M., Forn I., Gadelha C., Egan M.J., Bass D., Massana R., Richards T.A. (2011). Discovery of novel intermediate forms redefines the fungal tree of life. Nature.

[4] Kwong-Chung K.J., Bennett J.E. (1992). Medical Mycology.

[5] Boerema G.H., Bollen G.J. (1975). Conidiogenesis and conidial septation as differentiating criteria between *Phoma* and *Ascochyta*. Persoonia.

[6] Boerema G.H., de Gruyter J., Noordeloos M.E., Hamers M.E.C. (2004). Phoma Identification Manual. Differentiation of Specific and Infra-Specific Taxa in Culture.

[7] Guégan S., Garcia-Hermoso D., Sitbon K., Ahmed S., Moguelet P., Dromer F., Lortholary O., French Mycosis Study Group (2016). Ten-Year Experience of Cutaneous and/or Subcutaneous Infections Due to Coelomycetes in France. Open Forum Infect. Dis..

[8] Choi J.J., Kim S.H. (2017). A genome tree of life for the fungi kingdom. Proc. Natl. Acad. Sci. USA.

[9] Aveskamp M.M., de Gruyter J., Crous P.W. (2008). Biology and recent developments in the systematics of *Phoma*, a complex genus of major quarantine significance. Fungal Divers..

[10] Young N.A., Kwon-chung K.J., Freeman J. (1973). Subcutaneous abscess caused by *Phoma* sp. resembling *Pyrenochaeta romeroi*: Unique fungal infection occurring in immunosuppressed recipient of renal allograft. Am. J. Clin. Pathol..

[11] Nucci M., Queiroz-Telles F., Tobón A.M., Restrepo A., Colombo A.L. (2010). Epidemiology of opportunistic fungal infections in Latin America. Clin. Infect. Dis..

[12] Weidenbörner M., Hindorf H. (1989). Fungi isolated from protein enriched seeds and pods with special emphasis on the genus *Aspergillus*. Sci. Technol..

[13] Pitt J.I., Hocking A.D., Bhudhasamai K., Miscamble B.F., Wheeler K.A., Tanboon-Ek P. (1993). The normal mycoflora of commodities from Thailand. 1. Nuts and oilseeds. Int. J. Food Microbiol..

[14] Huang L.H., Hanlin R.T. (1975). Fungi occurring in freshly harvested and in-market pecans. Mycologia.

[15] Joffe A.Z. (1969). The mycoflora of fresh and stored groundnut kernels in Israel. Mycopathol. Mycol. Appl..

[16] Ko¨vics G.J., de Gruyter J., van der Aa H.A. (1999). *Phoma sojicola* comb. nov. and other hyaline-spored coelomycetes pathogenic on soybean. Mycol. Res..

[17] Temorshuizen A.J. (2007). Fungal and fungus-like pathogens of potato. Potato Biology and Biotechnology.

[18] Paterson R.R.M., Lima N. (2017). Filamentous Fungal Human Pathogens from Food Emphasising *Aspergillus*, *Fusarium* and *Mucor*. Microorganisms.

[19] Oliveira R.C., Goncalves S.S., Oliveira M.S., Dilkin P., Mallmann C.A., Freitas R.S., Bianchi P., Correa B. (2017). Natural occurrence of tenuazonic acid and *Phoma sorghina* in Brazilian sorghum grains at different maturity stages. Food Chem..

[20] Do Amaral A.L., de Carli M.L., Neto J.F.B., Dal Soglio F.K. (2004). *Phoma sorghina*, a new pathogen associated with *Phaeosphaeria* leaf spot on maize in Brazil. Plant Pathol. J..

[21] Adekoya I., Obadina A., Adaku C.C., De Boevre M., Okoth S., De Saeger S., Njobeh P. (2018). Mycobiota and co-occurrence of mycotoxins in South African maize-based opaque beer. Int. J. Food Microbiol..

[22] Kim G.H., Kim D.R., Park S., Lee Y.S., Jung J.S., Koh Y.J. (2017). Incidence rates of major diseases of kiwiberry in 2015 and 2016. Plant Pathol. J..

[23] Migheli Q., Cacciola S.O., Balmas V., Pane A., Ezra D., Magnano di San Lio G. (2009). Mal secco disease caused by *Phoma tracheiphila*: A potential threat to lemon production worldwide. Plant Dis..

[24] Kubota M., Kishi K., Abiko K. (2000). *Phoma* leaf spot, stem and fruit rot of tomato caused by *Phoma lycopersici* Cooke in Japan. Jpn. J. Phytopathol..

[25] Laundon G.F. (1971). Records of fungal plant disease in New Zealand. N. Z. J. Bot..

[26] Palavouzis S., Tzamos S., Paplomatas E., Thomidis T. (2015). First report of *Phoma Aliena* causing fruit rots of pomegranates in northern Greece. J. Plant Pathol..

[27] Cole R.J., Jarvis B.B., Schweikert M.A. (2003). Handbook of Secondary Fungal Metabolites.

[28] Lugauskas A., Raila A., Railiene M., Raudoniene V. (2006). Toxic micromycetes in grain raw material during its processing. Ann. Agric. Environ. Med..

[29] Weidenbörner M. (2001). Pine nutes: The mycobiota and potential mycotoxins. Can. J. Microbiol..

[30] Visconti A., Logrieco A., Vurro M., Bottalico A. (1987). Tenuazonic acid in blackmold tomatoes: Occurrence, production by associated *Alternaria* species, and phytotoxic properties. Phytopathol. Mediterran..

[31] Yekeler H., Bitmis K., Özcelik N., Doymaz M.Z., Metin C. (2001). Analysis of toxic effects of *Alternaria* toxins on esophagus of mice by light and electron microscopy. Toxicol. Pathol..

[32] Sutton D.A., Rinaldi M.G., Sanche S.E., Anaissie E.J., McGinnis M.R., Pfaller M.A. (2009). Dematiaceous fungi. Clinical Mycology.

[33] Rai M.K., Tiwari V.V., Irinyi L., Kövics G.J. (2014). Advances in taxonomy of genus *Phoma*: Polyphyletic nature and role of phenotypic traits and molecular systematics. Indian J. Microbiol..

[34] Valenzuela-Lopez N., Sutton D.A., Cano-Lira J.F., Paredes K., Wiederhold N., Guarro J., Stchigel A.M. (2017). Coelomycetous Fungi in the Clinical Setting: Morphological Convergence and Cryptic Diversity. J. Clin. Microbiol..

[35] Chen Q., Jiang J.R., Zhang G.Z., Cai L., Crous P.W. (2015). Resolving the *Phoma* enigma. Stud. Mycol..

[36] Babič M.N., Gunde-Cimerman N., Vargha M., Tischner Z., Magyar D., Veríssimo C., Sabino R., Viegas C., Meyer W., Brandão J. (2017). Fungal Contaminants in Drinking Water Regulation? A Tale of Ecology, Exposure, Purification and Clinical Relevance. Int. J. Environ. Res. Public Health.

[37] Doggett M.S. (2000). Characterization of Fungal Biofilms within a Municipal Water Distribution System. Appl. Environ. Microbiol..

[38] Aho R., Hirn J. (1981). A survey of fungi and some indicator bacteria in chlorinated water of indoor public swimming pools. Zentralbl. Bakteriol. Mikrobiol. Hyg. B.

[39] Ekowati Y., van Diepeningen A.D., Ferrero G., Kennedy M.D., de Roda Husman A.M., Schets F.M. (2017). Clinically relevant fungi in water and on surfaces in an indoor swimming pool facility. Int. J. Hyg. Environ. Health.

[40] Hamada N., Abe N. (2010). Growth characteristics of four fungal species in bathrooms. Biocontrol. Sci..

[41] Zhang T., Wang N.F., Zhang Y.Q., Liu H.Y., Yu L.Y. (2016). Diversity and Distribution of Aquatic Fungal Communities in the Ny-Ålesund Region, Svalbard (High Arctic): Aquatic Fungi in the Arctic. Microb. Ecol..

[42] Calvo M.A., Guarro J., Suarez G., Ramírez C. (1980). Air-borne fungi in the air of Barcelona (Spain). IV. Various isolated genera. Mycopathologia.

[43] A’Hara D. (2015). Detection and identification of *Phoma* pathogens of potato. Methods Mol. Biol..

[44] Food and Agriculture Organization (2013). Revised Guidelines for the Assessment of Microbiological Quality of Processed Foods. http://fda.gov.ph./attachments/article/17218/FC2013-010.pdf.

[45] Bandyopadhyay R., Mughogho L.K., Satyanarayana M.V., Kalisz M.E. (1991). Occurrence of airborne spores of fungi causing grain mould over sorghum crop. Mycol. Res..

[46] González H.H., Martínez E.J., Resnik S.L. (1997). Fungi associated with sorghum grain from Argentina. Mycopathologia.

[47] Hussaini A.M., Timothy A.G., Olufunmilayo H.A., Ezekiel A.S., Godwin H.O. (2009). Fungi and some mycotoxins found in mouldy Sorghum in Niger state, Nigeria. World J. Agric. Sci..

[48] (2010–2017). Food and Agriculture Organization of the United Nations. https://www.fao.org/giews/countrybrief/country.jsp?code=BRA.

[49] Janke D. (1956). About a human-pathogenic new species of *Peyronellaea* bred from lung changes. Mycopathologia.

[50] Bakerspigel A. (1970). The isolation of *Phoma hibernica* from lesions on a leg. Sabouraudia.

[51] Bakerspigel A., Lowe D., Rostas A. (1981). The isolation of *Phoma eupyrena* from a human lesion. Arch. Dermatol..

[52] Roehm C.E., Salazar J.C., Hagstrom N., Valdez T.A. (2012). *Phoma* and *Acremonium* invasive fungal rhinosinusitis in congenital acute lymphocytic leukemia and literature review. Int. J. Pediatr. Otorhinolaryngol..

[53] Gordon M.A., Salkin I.F., Stone W.B. (1975). *Phoma (Peyronellaea)* as zoopathogen. Sabouraudia.

[54] Punithalingam E. (1979). Sphaeropsidales in culture from humans. Nova Hedwig..

[55] Shukla N.P., Rajak R.K., Agarwasl G.P., Gupta D. (1984). *Phoma minutispora* as a human pathogen. Mykosen.

[56] Baker J.G., Salkin I.F., Forgacs P., Haines J.H., Kemna M.E. (1987). First report of subcutaneous phaeohyphomycosis of the foot caused by *Phoma minutella*. J. Clin. Microbiol..

[57] Rai M.K. (1989). *Phoma sorghina* infection in human being. Mycopathologia.

[58] Hirsh A.H., Schiff T.A. (1996). *Subcutaneous phaeohyphomycosis* caused by an unusual pathogen: *Phoma* species. J. Am. Acad. Dermatol..

[59] Rosen T., Rinaldi M.G., Tschen J.A., Stern J.K., Cernoch P. (1996). Cutaneous lesions due to *Pleurophoma* (*Phoma*) Complex. South Med. J..

[60] Zaitz C., Heins-Vaccari E.M., de Freitas R.S., Arriagada G.L., Ruiz L., Totoli S.A., Marques A.C., Rezze G.G., Müller H., Valente N.S. (1997). *Subcutaneous phaeohyphomycosis* caused by *Phoma cava*. Report of a case and review of the literature. Rev. Inst. Med. Trop. Sao Paulo.

[61] Arrese J.E., Piérard-Franchimont C., Piérard G.E. (1997). Unusual mould infection of the human stratum corneum. J. Med. Vet. Mycol..

[62] Oh C.K., Kwon K.S., Lee J.B., Jang H.S., Chung T.A., Suh S.B. (1999). *Subcutaneous phaeohyphomycosis* caused by *Phoma* species. Int. J. Dermatol..

[63] Everett J.E., Busick N.P., Sielaff T., Wahoff D.C., Dunn D.L. (2003). A deeply invasive *Phoma* species infection in a renal transplant recipient. Transplant. Proc..

[64] Suh M.K. (2005). Phaeohyphomycosis in Korea. Nihon Ishinkin Gakkai Zasshi.

[65] Vasoo S., Yong L.K., Sultania-Dudani P., Scorza M.L., Sekosan M., Beavis K.G., Huhn G.D. (2011). Phaeomycotic cysts caused by *Phoma* species. Diagn. Microbiol. Infect. Dis..

[66] Schieffelin J.S., Garcia-Diaz J.B., Loss G.E., Beckman E.N., Keller R.A., Staffeld-Coit C., Garces J.C., Pankey G.A. (2014). Phaeohyphomycosis fungal infections in solid organ transplant recipients: Clinical presentation, pathology, and treatment. Transpl. Infect. Dis..

[67] Hernández-Hernández F., Vargas-Arzola J., Ríos-Cruz O.P., Córdova-Martínez E., Manzano-Gayosso P., Segura-Salvador A. (2018). First case of chromoblastomycosis due to *Phoma insulana*. Enferm. Infecc. Microbiol. Clin..

[68] Rishi K., Font R.L. (2003). Keratitis caused by an unusual fungus, *Phoma* species. Cornea.

[69] Errera M.H., Barale P.O., Nourry H., Zamfir O., Guez A., Warnet J.M., Sahel J.A., Chaumeil C. (2008). Usefulness of voriconazole in treatment of *Phoma glomerata* after penetrating injury. J. Fr. Ophthalmol..

[70] Kumar P., Thomas S., Papagiannuli E., Hardman S.C., Jenkins D., Prydal J. (2015). A case of *Phoma* fungal keratitis in a contact lens user. JRSM Open.

[71] McElnea E., Farrell S., Lynch B., Bishop K., Mullen D., Borman A., Higgins G. (2015). A rare case of fungal keratitis: Diagnosis and management. JMM Case Rep..

[72] Morris J.T., Beckius M.L., Jeffery S., Longfeld R.N., Heaven R.F., Baker W.J. (1995). Lung mass caused by *Phoma* species. Infect. Dis. Clin. Pract..

[73] Balis E., Velegraki A., Fragou A., Pefanis A., Kalabokas T., Mountokalakis T. (2006). Lung mass caused by *Phoma exigua*. Scand. J. Infect. Dis..

[74] Tullio V., Banche G., Allizond V., Roana J., Mandras N., Scalas D., Panzone M., Cervetti O., Valle S., Carlone N. (2010). Non-dermatophyte moulds as skin and nail foot mycosis agents: *Phoma herbarum, Chaetomium globosum* and *Microascus cinereus*. Fungal Biol..

[75] Punithalingam E. (1976). *Phoma oculohominis* sp. nov. from corneal ulcer. Trans. Br. Mycol. Soc..

[76] Chowdhary A., Meis J.F., Guarro J., de Hoog G.S., Kathuria S., Arendrup M.C., Arikan-Akdagli S., Akova M., Boekhout T., Caira M. (2014). ESCMID and ECMM joint clinical guidelines for the diagnosis and management of systemic phaeohyphomycosis: Diseases caused by black fungi. Clin. Microbiol. Infect..

[77] Sutton D.A. (1999). Coelomycetous fungi in human disease. A review: Clinical entities, pathogenesis, identification and therapy. Rev. Iberoam. Micol..

[78] Brenier-Pinchart M.P., Faure O., Garban F., Fricker-Hidalgo H., Mallaret M.R., Trens A., Lebeau B., Pelloux H., Grillot R. (2006). Ten-year surveillance of fungal contamination of food within a protected haemotological unit. Mycoses.

